# New School of Scientific Medicine

**Published:** 1898-09

**Authors:** 


### NEW SCHOOL OF SCIENTIFIC MEDICINE.

Surgeon-General Sternberg, in his recent address, as president, before the American Medical Association at Denver, repudiates the title of "*Old School*" as applied to the fast filling ranks of scientific thinkers in the medical profession, and substitutes another name, that of "*The New School of Scientific Medicine*." This name was not original with the surgeon-general and president of the American Medical Association, but was voiced years ago in the columns of the *Medical Times* and was received with sneers by the so-called "Old School," and a howl of indignation, misrepresentation and abuse by many of the journals and would-be leaders of the new school, all of which contended that the time had not yet come for a union in work and investigation under the folds of one flag. More than ten years ago one of the editors of this journal gave notice at a meeting of the American Institute of Homeopathy that at its next meeting he intended to move that the name of the Institute be changed to that of the "*American Institute of Scientific Medicine*," basing his reasons for the change that the trend of scientific investigation in all schools was along the same lines and was reaching conclusions so impregnable as to elevate the profession from the domain of empiricism, with its warring factions, to the ranks of science. "Much as the 'new school' had accomplished in the way of scientific medicine, and proudly as it might point to its records of the practical good it had accomplished and the change it had produced in medical thought and practice throughout the world, it was hampered and its usefulness impaired by a sectarian name, which was very far from voicing or defining its actual work. We are physicians pledged to one of the noblest duties to which humanity can be called, upon the proper fulfillment of which depends to a certain extent not only the welfare of the body, but of the soul. It is ours to penetrate with the light of science the dark chambers of disease, to cleanse the channels of life and say to the 'pestilence which wasteth at noonday,' 'Thus far but no farther.' The time has come for a union along scientific lines, for independent thought, for free investigation in which no creed or pathy shall be permitted to block the way. This school which has done so much for humanity, can well afford to take the initiative in a movement which sooner or later will dominate the medical world."

This proposition was received with such a storm of derision that no future effort was made to carry it into effect. The *Medical Times*, however, has kept steadily on with its work, firmly believing in the correctness of its position and without a single doubt of its ultimate triumph. Scarcely a decade has passed when the Surgeon-General of the United States advances, as President of the American Medical Association, gathered at Denver from every State in the Union, with all the weight of the medical head of an army whose heroic deeds are now thrilling the hearts of civilized nations throughout the world, precisely the same idea presented in the American Institute of Homeopathy, backed by almost the same argument. Indeed, that portion of General Sternberg's address, in which he claims that the term "old school" is entirely inappropriate and insists that for a profession which, as a whole, within the past few years has been moving forward with such incredible activity upon the substantial basis of scientific research, if characterized by any distinctive name, it should be the "*New School of Scientific Medicine*," is almost precisely in spirit and in language like the editorials which have so often appeared in the *Times*.

The emphatic assertion that "there is now no room for creed and pathies in medicine any more than in astronomy, geology, or botany, and that every man is entitled to his own opinion upon any unsettled question, and that no restriction should be placed upon any regular educated physician, as to his mode of treatment in any given case," did not meet with the endorsement of all in the society. When the motion of Dr. Hare, the distinguished author and teacher, was presented, "That this Association invites the New York State Medical Society, the New York Academy of Medicine and other societies of good and regular standing to send delegates to this association," it was too much for one of the members, Dr. Munn, of Denver, who denounced the resolution as the inoculated scalpel by which it is proposed to introduce the sepsis of commercialism into the association. "In this Western country," said Dr. Munn, "we have had all we could do to keep out the dead rot of commercialism, and are we going to be trodden down by this derelict, dead, rotten society of New York, which is continually sending——." A storm of hisses rendered the final words of the closing sentence inaudible. Every one has heard of Dr. Hare; his works are found in almost every medical library, but who has heard of Dr. Munn? What have the societies mentioned in Dr. Hare's resolution done to be characterized as dead and rotten, and held up to the contempt of the American Medical Association by Dr. Munn and his confreres? Simply broken loose from an association in this State controlled by an iron-bound code of ethics, and established one with no code of ethics but that of the gentleman. These societies are composed of some of the most advanced men of the State, who have stepped out of the thraldom of the sixteenth century into the light and freedom of an age teeming with life and progress. Possibly the American Medical Association will learn, at no distant day, that the walls of truth stand, but the walls of denominational separation are crumbling. Let the standard of the new school of scientific medicine be unfurled, and the thoughtful, unprejudiced minds of all schools will rally to its support.—*New York Medical Times.*

